# A Revised Model of Trust in Internet-Based Health Information and Advice: Cross-Sectional Questionnaire Study

**DOI:** 10.2196/11125

**Published:** 2019-11-11

**Authors:** Elizabeth Sillence, John Matthew Blythe, Pam Briggs, Mark Moss

**Affiliations:** 1 Psychology and Communication Technology Lab Department of Psychology Northumbria University Newcastle upon Tyne United Kingdom; 2 Dawes Centre for Future Crime UCL Jill Dando Institute of Security and Crime Science University College London London United Kingdom; 3 Department of Psychology Northumbria University Newcastle upon Tyne United Kingdom

**Keywords:** trust, eHealth, patient experiences

## Abstract

**Background:**

The internet continues to offer new forms of support for health decision making. Government, charity, and commercial websites increasingly offer a platform for shared personal health experiences, and these are just some of the opportunities that have arisen in a largely unregulated arena. Understanding how people trust and act on this information has always been an important issue and remains so, particularly as the design practices of health websites continue to evolve and raise further concerns regarding their trustworthiness.

**Objective:**

The aim of this study was to identify the key factors influencing US and UK citizens’ trust and intention to act on advice found on health websites and to understand the role of patient experiences.

**Methods:**

A total of 1123 users took part in an online survey (625 from the United States and 498 from the United Kingdom). They were asked to recall their previous visit to a health website. The online survey consisted of an updated general Web trust questionnaire to account for personal experiences plus questions assessing key factors associated with trust in health websites (information corroboration and coping perception) and intention to act. We performed principal component analysis (PCA), then explored the relationship between the factor structure and outcomes by testing the fit to the sampled data using structural equation modeling (SEM). We also explored the model fit across US and UK populations.

**Results:**

PCA of the general Web trust questionnaire revealed 4 trust factors: (1) personal experiences, (2) credibility and impartiality, (3) privacy, and (4) familiarity. In the final SEM model, trust was found to have a significant direct effect on intention to act (beta=.59; *P*<.001), and of the trust factors, only credibility and impartiality had a significant direct effect on trust (beta=.79; *P*<.001). The impact of personal experiences on trust was mediated through information corroboration (beta=.06; *P*=.04). Variables specific to electronic health (eHealth; information corroboration and coping) were found to substantially improve the model fit, and differences in information corroboration were found between US and UK samples. The final model accounting for all factors achieved a good fit (goodness-of-fit index [0.95], adjusted goodness-of-fit index [0.93], root mean square error of approximation [0.50], and comparative fit index [0.98]) and explained 65% of the variance in trust and 41% of the variance in intention to act.

**Conclusions:**

Credibility and impartiality continue to be key predictors of trust in eHealth websites. Websites with patient experiences can positively influence trust but only if users first corroborate the information through other sources. The need for corroboration was weaker in the United Kingdom, where website familiarity reduced the need to check information elsewhere. These findings are discussed in relation to existing trust models, patient experiences, and health literacy.

## Introduction

### Background

The number of people using the internet for health information and advice continues to grow with people affected by long-term or chronic conditions making particular use of online resources [1]. Over 80% of teens have sought health information online at some point about a range of health and lifestyle issues [2], and there has been a rise in surrogate seekers, those seeking information online for someone else [3]. Understanding how people come to trust the information and advice they find online has been an important issue since the widespread adoption of the internet [4] and continues to be so (see, eg, recent work by Marcu et al [5] and Lu et al [6]). The explosion in new providers, new formats, and platforms continues to generate concerns regarding the quality and variability of the health information available to the average citizen. Despite the introduction of codes and standards, for example, *Health on the Internet* code, early concerns over information quality, accuracy, and credibility [7] are still being echoed by researchers examining the provision of electronic health (eHealth) material across a range of conditions [8] including diabetes, osteoarthritis, and orthognathic surgery [9-11]. Today, such concerns sit within a wider debate about the veracity of information available to citizens through a variety of online sources. We know that people will often make snap judgments about the quality of information available online [4,12], relying upon simple heuristics to inform their decision making. We also know that people seldom make these judgments in isolation but are likely to show social influences in their information searches [12]. In particular, we can see that citizens exhibit homophily when going online for information—choosing to be guided by others they perceive as similar to themselves [13] and selecting information that is consistent with their own prior beliefs [14].

These social effects are particularly strong when people share their own health experiences online. Shared personal experiences are important to health consumers [15,16], and these are disseminated in online support communities, which can offer long-term supportive relationships, providing empathy, and reducing patients’ sense of isolation [17,18]. As online social networks have grown, the range and availability of personal experiences have grown enormously. Peer-to-peer resources in the form of support forums, blogs, written or video testimonials, as well as curated experiences have become a common feature of online health resources. They are found in eHealth sites provided not just by concerned individuals but by charities, governmental organizations, and commercial websites alike. They once again put the concept of a trusting relationship center stage as the mediating technology, the host platform, and the contributors themselves can all be considered as objects of trust [19]. Put simply, a health consumer must typically make a number of layered trust decisions before engaging with peer-led material on a site [13], although a credible *host* site may be a prerequisite for trust in the more personal stories or blogs contained within [20].

The sheer number of eHealth resources available means that there are significant opportunities to check and verify any information and advice found online. Indeed, corroboration has been shown to have a key role in predicting trust and action around eHealth information [21]. However, research indicates that once again we see different factors influencing the layered trust decisions that are made [21]. At the peer-led level, when people are seeking to check information about personal experiences, these corroborating activities may become distorted by social networks, where the homophily effects of being able to tap into information bubbles of “people like me” may act to limit the effectiveness of cross-checking, particularly for groups with low socioeconomic status (eg, [14]). At the platform or website level, other factors come into play. Thus, for example, many websites require commercial funding, and this in turn can be signaled by the presence of online advertising, which in turn may act to undermine the perceived trustworthiness of the messages on the site. Impartiality is fundamental to trust in online resources [22], and advertising can lead a consumer to question the underlying motivations of an organization, sensing that they may not necessarily be acting primarily in the interests of a patient or carer [23]. Genuine peer contributors to a forum or site may wish to convey a credible, persuasive account of their experience with a particular product or service, but, if the narrative is framed in a commercial context, then the veracity of that experience may be called into question [24,25]. In general, it appears that personal experiences and commerce do not work well together. The blurring of the lines between testimonials and advertising serves to reduce the value of the personal accounts and the overall credibility of the website [23]. Furthermore, new trust concerns arise for both contributors and consumers of health content if people feel that the information they provide or access may be used to profile their own health status. This is a critical issue in the wake of new developments in the United States that give more freedom to internet service providers to sell on consumer information to advertisers [26], making health privacy a critical, but as yet under-researched aspect of trust decision making in eHealth [27].

Understanding the antecedents of trust in online health information has been a long-standing interest of the authors who, for the past 20 years, have developed and reported a number of large-scale eHealth surveys to gauge changes in the trust practices of people seeking health information online. Taken together, the studies have addressed the rise in patient-centered and patient-generated health information.

Since 2000, the range of patient-led resources and the nature and number of different eHealth providers have grown dramatically, and the most recent changes have seen a dramatic rise in patient narratives, often accompanied by new advertising funding models that may not always be viewed as appropriate in a health domain. The noticeable shift toward the inclusion of peer-led information creates interesting questions around what exactly it is that we are being asked to trust—the advice, the patient who provides a *story*, the organization behind the website, or other (sometimes unknown) funders. All of these can influence the decision to trust, and subsequently act upon, health advice. The extent to which *health privacy* affects trust in eHealth is also poorly understood. It is, therefore, timely to ask again about how people make their trust decisions.

Data we collected 10 years ago [21] resulted in a model that showed how trust in eHealth information and intention to act on the advice could be predicted on the basis of source credibility and impartiality. In that study, the predictive value of these 2 factors was enhanced when consumer responses to uncongenial health-risk information was taken into account. In particular, adding variables specific to health psychology (eg, measures addressing coping style), alongside measures designed to capture response to the online environment (eg, information corroboration), enhanced the model’s predictive power.

### Objectives

In this study, we aimed to update this model and provide a more timely understanding of the current antecedents of trust in online health information. We did this in 2 steps. First, we assessed the factorial structure of an updated general measure of trust in online health resources. We took the general measure of trust used in the study by Harris et al [21] and supplemented it with measures addressing inter alia personal experiences online, the presence of advertising, and health privacy concerns. Second, we sought to establish how well these subsequent factors improved the predictive power of the older model [21]. In addition, we purposely sampled from the United States and the United Kingdom to establish the robustness of the model across 2 widely different health care economies, one largely privatized (with funding via a complex health insurance network) and the other largely nationalized.

In summary, then, we sought to model the role of online personal experiences in health information and advice-seeking behavior using populations drawn from the United States and the United Kingdom.

## Methods

### Design

A cross-sectional survey was conducted in November 2015 and collected quantitative data from eHealth users regarding their use of health websites as part of a larger project measuring online trust in health websites every 5 years since 2000. We used a panel company to recruit a similar demographic to those that had participated in our previous studies to gain a sample representative enough to allow for meaningful comparisons.

### Participants

A total of 8272 people clicked on the link from the recruitment advertisement on the panel company’s internal Web page and were assessed on their eligibility to take part in the survey. Of this larger sample, 74.62% (6172/8272) indicated that they used the internet to look for health advice compared with 25.43% (2103/8272) who did not use the internet for advice. Following eligibility assessment (older than 18 years and UK- [40% quota] or US-based [60% quota]), a total of 1396 participants completed the questionnaire. A total of 96 were removed because of incomplete data resulting in 1123 participants that completed the full survey exploring online health seekers. Of the 1123 participants, 875 (77.92%) reported searching the internet for health advice for themselves, 145 (12.91%) for someone else, and the remaining 112 (9.97%) for both. Participants received £1.71 (or the US equivalent) for taking part in the study. Full details of participant demographics can be found in Table 1.

**Table 1:** Participant demographics (N=1123).

Participant characteristic			Online seekers frequency, n (%)
**Location**			
	United States	625 (55.65)	
	United Kingdom	498 (44.34)	
**Gender**			
	Male	462 (41.14)	
	Female	661 (58.86)	
**Age (years)**			
	18-24	172 (15.32)	
	25-35	311 (27.69)	
	36-44	222 (19.77)	
	45-54	195 (17.36)	
	55-64	146 (13.00)	
	65+	63 (5.61)	
**Employment status**			
	Full time	545 (48.53)	
	Part time	171 (15.23)	
	Retired	137 (12.20)	
	Unemployed	208 (18.52)	
	Student	62 (5.52)	
**Marital status**			
	Single	354 (31.52)	
	Married	531 (47.28)	
	Cohabiting	106 (9.44)	
	Civil partnership	29 (2.58)	
	Divorced	84 (7.48)	
	Widowed	19 (1.69)	
**Ethnicity**			
	White	912 (81.21)	
	Latino/Hispanic	40 (3.56)	
	Middle Eastern	12 (1.07)	
	African	59 (5.25)	
	Caribbean	11 (0.98)	
	South Asian	23 (2.05)	
	East Asian	20 (1.78)	
	African American	11 (0.98)	
	Mixed	18 (1.60)	
	Prefer not to say	16 (1.42)	
**Highest level of education**			
	Less than high school/secondary school	18 (1.60)	
	Secondary school/high school/general educational development	294 (26.18)	
	Further education (college, A-levels or equivalent)	199 (17.72)	
	Bachelor’s degree	490 (43.63)	
	Postgraduate degree (MSc, PhD)	122 (10.86)	
**Internet use (years)**			
	1-2	7 (0.62)	
	3-5	46 (4.10)	
	6-9	98 (8.73)	
	10-14	313 (27.87)	
	15-19	350 (31.17)	
	20+	309 (27.52)	

### Procedure

Before study commencement, the study received full ethical approval from the Department of Psychology at Northumbria University, and the online survey was piloted with 5 participants to assess comprehension and running of the survey. The survey was hosted on Qualtrics. The first page provided participants with information detailing the aim, length, data storage, contact details, and withdrawal process of study. They were then asked to provide informed consent. The study then commenced, and participants were asked whether they used the internet to look for health advice. Those answering “yes” then completed a series of questions relating to the last time they searched for health advice online. Specifically, they were asked to “think about any one site that you visited during that search” and to answer the remaining questions with respect to that site. They answered questions relating to the impact of the health advice on their coping perceptions and intention to act, the degree to which they trusted the information and website, and demographic information.

### Measures

Unless stated otherwise, participants answered the following measures on a 5-point Likert scale (*1=strongly disagree* to *5=strongly agree*).

#### General Web Trust Questionnaire

The first measure contained the 24 items from the study by Harris et al [21], supplemented by 8 items assessing the presence of personal experiences [28] and 5 items to measure privacy concerns. In addition, *coping* was measured with 4 items such as “Looking at this site made me feel in control,” in which participants’ responses were rated on a 6-point scale with the following labels: *1=less, 2=slightly less, 3=no different, 4=slightly more, and 5=more* (Cronbach alpha=.83). *Information corroboration* with other sources of information was measured with the following 2 items: (1) “I checked other websites” and (2) “I checked other sources” (Cronbach alpha=.85). It is recognized that having just 2 items contributing to a measure can give challenge to the accuracy of Cronbach alpha, although in such cases alpha acts as a lower bound for the reliability, that is, it always underestimates the true reliability of the scale [29]. Note that these items were all taken from an earlier study [21].

#### Outcome Measures

*Trust* was measured with the following 2 items: (1) “I trusted the site” and (2) “I felt I could trust the information on the site” (Cronbach alpha=.78). *Intention to act* was an outcome measure, assessed with 1 item “I intended to act upon the advice.”

## Results

We first explored the updated general Web trust questionnaire by performing principal component analysis (PCA). We then explored the relationship between the factor structure and outcomes by testing its fit to the sampled data using structural equation modeling (SEM).

### Properties of the General Web Trust Questionnaire

The 36 items of the scale were entered into the PCA, and varimax rotation with Kaiser normalization was used. Any items with factor loadings lower than 0.30 were suppressed (see Table 2).

The findings from the PCA revealed that 4 components (with eigenvalues above 1) could explain the data accounting for 66.057% of the variance. This complied with the minimum acceptable level of 60% variance and recommendations of eigenvalues above 1 for factors [30]. One item *The site was free from advertisements* did not load onto any component and was dropped from the analysis. In other words, this component was not, in isolation, a strong enough measure to be considered influential in the final model.

Overall, the analysis revealed that the 4 final components explained a large amount of the variance in the data, and the items had strong component loadings (well above the 0.30 criterion). It is recognized that the fourth component could be considered as weak as it only comprises 2 items. Advice is that there should be a minimum of 3 items per extracted component. However, it is reasonable that a component with 2 items is *identified*, provided that the other factors have more than 3 items and the 2-item factor has a nonzero covariance with at least one other factor in the population [31]. Such is the case here.

**Table 2:** Factor loadings for each item (factor loadings lower than 0.30 are suppressed).

Item	Rotation factor loadings			
	Factor 1: Personal experiences^a^	Factor 2: Credibility and impartiality^b^	Factor 3: Privacy^c^	Factor 4: Familiarity^d^
The language on the site made it easy to understand.	—^e^	0.783	—	—
The site helped me understand the issue better.	—	0.791	—	—
The site was easy to use.	—	0.780	—	—
The site told me most of what I needed to know.	—	0.692	—	—
The layout was consistent with other sites.	—	0.608	—	—
The advice appeared to be prepared by an expert.	—	0.664	—	—
The advice seemed to be offered in my best interests.	—	0.744	—	—
The advice came from a knowledgeable source.	—	0.714	—	—
The advice seemed credible.	—	0.747	—	—
The site was owned by a well-known organization.	—	—	—	0.769
The site featured familiar logos.	—	—	—	0.795
The site had a professional design.	—	0.679	—	—
The site had an attractive design.	—	0.605	—	—
The site provided reassurances about my privacy.	—	—	0.616	—
The site gave the option to post anonymously.	—	—	0.669	—
The site gave reassurances about how they used your information.	—	—	0.739	—
The site had a privacy policy.	—	—	0.717	—
The site explained their use of cookies.	—	—	0.637	—
The site contained accounts of other patient experiences.	0.815	—	—	—
There was a chance to share my experiences.	0.821	—	—	—
There were opportunities to interact with other people on the site.	0.829	—	—	—
On the site I saw a wide range of experiences rather different to mine.	0.791	—	—	—
The site offered powerful accounts of health experiences.	0.817	—	—	—
It felt like the advice was tailored to me personally.	0.559	—	—	—
On the site, I was offered the chance to see experiences from people just like me.	0.856	—	—	—
The site contained contributions from like-minded people.	0.863	—	—	—
I was able to contribute to content on the site.	0.817	—	—	—
The personal accounts on the site were written by people similar to me.	0.882	—	—	—
I found personal accounts that reflected my own experience.	0.875	—	—	—
I found personal accounts that were relevant to my condition.	0.876		—	—
There were opportunities to gather information from the personal accounts on the site.	0.870	—	—	—
The personal accounts contained advice for readers.	0.869	—	—	—
The personal accounts provided social or emotional support.	0.845	—	—	—
The advice appeared to be impartial and independent.	—	0.682	—	—
The advice seemed objective (ie, no hidden agenda).	—	0.695	—	—
Removed item (the site was free from advertisements).	—	—	—	—

^a^Eigenvalue for factor 1 was 10.849, and the variance explained was 30.998%.

^b^Eigenvalue for factor 2 was 7.432, and the variance explained was 21.234%.

^c^Eigenvalue for factor 3 was 3.158, and the variance explained was 3.158%.

^d^Eigenvalue for factor 4 was 1.681, and the variance explained was 1.681%.

^e^Not applicable.

### Structural Equation Modeling Analysis

The data were analyzed using SEM performed in IBM SPSS AMOS and based on the model structure from Harris et al [21], which represents the data collected 10 years ago. Maximum likelihood estimation methods were used to assess model fit, and the input for each analysis was the covariance matrix of the items. The goodness-of-fit for the models was evaluated with the following absolute goodness-of-fit indices (GFIs) [32]: (1) the Chi-square goodness-of-fit statistic; (2) the root mean square error of approximation (RMSEA); (3) GFI; (4) the adjusted goodness-of-fit (AGFI), and (5) comparative fit index (CFI). Nonsignificant Chi-square values indicate that the hypothesized model fits the data, and RMSEA values smaller than or equal to 0.08 are indicative of acceptable fit. However, values above 0.1 should lead to model rejection [33]. GFI values greater than 0.95 are indicative of good fit, and values greater than 0.90 are indicative of an acceptable fit [34]. AGFI values of 0.90 are indicative of a good fit, and values greater than 0.85 may be considered an acceptable fit [35]. The closer the CFI value is to 1 the better the fit [36].

The final model accounted for 65% of the variance in trust, 27% of the variance in coping, and 41% of the variance in intention to act. The model was a good fit for 4 of the indices. The fit indices for GFI and AGFI were 0.95 and 0.93, which are indicative of a good fit. RMSEA was 0.050, and CFI was 0.98. Path coefficients (beta) and R^2^ values were also inspected in evaluating the predictive power of the models. Although the Chi-square indicated that the model was not a good fit to the data, Χ^2^_168_=639.8, *P*<.001, Chi-square has been criticized for being too sensitive to large sample sizes, especially for samples over 200 [37], as in this study.

Only credibility and impartiality was found to have a significant, direct relationship with trust (see Table 3). Familiarity and presence of personal experiences did not significantly relate to trust. The effects of familiarity, personal experiences, and privacy may be indirect and mediated through the other trust variables. In particular, personal experiences was found to have a significant direct effect on information corroboration, which in turn significantly predicted trust. Individuals who are presented with *personal experiences* may, therefore, corroborate this information with other sources and websites enhancing their trust in the personal experiences account. Trust in turn was found to significantly relate to coping perceptions and intention to act on the advice. This suggests that trustworthy websites heighten their coping perceptions, making them feel reassured, in control, and able to cope.

**Table 3:** The regression weights and critical ratio (ie, Z-score) values for the main effects of the hypothesized full model (combined UK and US participants).

Parameter	Unstandardized path coefficients	Critical ratio	*P* value
Credibility and impartiality → trust	0.944	17.110	<.001
Familiarity → trust	0.012	0.552	.58
PEX^a^ → trust	0.021	0.960	.34
Information corroboration → trust	0.050	3.001	.003
Credibility and impartiality → information corroboration	0.520	7.566	<.001
Familiarity → information corroboration	−0.051	−1.289	.20
PEX → information corroboration	0.067	2.092	.04
Trust → coping	2.229	16.518	<.001
Trust → intention to act	0.794	16.197	<.001
Coping → intention to act	0.013	1.425	.15
Information corroboration → intention to act	0.063	2.751	.006

^a^PEX: personal experiences.

### Comparison of Two Populations

A total of 2 further structural equation models were then assessed; one for each of the 2 populations that made up the full dataset, those from the United States and those from the United Kingdom. Although no previous literature exists to document consumer differences in terms of their trust in online health information, the countries differ widely in terms of state-run health provision, and it is known that health consumers differ in terms of their internet health behaviors [38] and that physicians in the United States and the United Kingdom differ widely in terms of their access to online information [39].

#### US Population

The model was a good fit for 4 of the indices. The GFI and AGFI were 0.93 and 0.91, respectively, and the RMSEA and CFI were 0.055 and 0.97, respectively, although the Chi-square indicated that the model was not a good fit to the data, Χ^2^_168_=481.3, *P*<.001 (see earlier above). Path coefficients (beta) and R^2^ values were also inspected in evaluating the predictive power of the models. The final model accounted for 64% of the variance in trust, 27% of the variance in coping, and 44% of the variance in intention to act. Regression weights are presented in Table 4 below.

**Table 4:** The regression weights and critical ratio values for the main effects of the hypothesized model for US participants.

Parameter	Unstandardized path coefficients	Critical ratio	*P* value
Credibility and impartiality → trust	1.001	13.346	<.001
Familiarity → trust	–0.052	–1.515	.13
PEX^a^ → trust	0.073	2.436	.02
Information corroboration → trust	0.068	3.023	.003
Credibility and impartiality → information corroboration	0.364	3.959	<.001
Familiarity → information corroboration	0.018	0.308	.76
PEX → information corroboration	0.060	1.408	.16
Trust → coping	2.224	12.696	<.001
Trust → intention to act	0.802	13.216	<.001
Coping → intention to act	0.008	0.651	.52
Information corroboration → intention to act	0.075	2.485	.01

^a^PEX: personal experiences.

There are 2 differences in the observed relationships when comparing the US model with the full model. First, the significant predictive path between personal experiences and information corroboration is lost. However, given that the regression weight is identical in both models, this is just a consequence of reduced power in the US analysis. More notable is the introduction of a significant path between personal experiences and trust that is not evident in the full model. All other paths are comparable between the 2 models.

#### UK Population

Although the Chi-square indicated that the model was not a good fit to the data, Χ^2^_168_=422.8, *P*<.001, the model was a good fit for the remaining 4 indices. The GFI and AGFI were 0.92 and 0.89, respectively. Finally, RMSEA was 0.055, and CFI was 0.97. Path coefficients (beta) and R^2^ values were also inspected in evaluating the predictive power of the models. The final model accounted for 65% of the variance in trust, 27% of the variance in coping, and 38% of the variance in intention to act. Regression weights are presented in Table 5.

As with the US-based model, the significant predictive path between personal experiences and information corroboration is lost. Equally, however, the regression weight is identical in both models, and this is just a consequence of reduced power in the UK analysis. A total of 2 further paths also fail to reach significance in the UK model compared with the full model: information corroboration to trust and to intention to act. For these 2, there is a noticeable reduction in the regression coefficients for the UK model compared with both the US and full models, and as such the loss of significance is a consequence of a weaker relationship as well as a reduction in power. Moreover, the UK model also produces a significant path between familiarity and information corroboration that is not present in either the full or the US model.

In summary, although the US- and UK-based analyses share—as might be expected—many of the significant relationships identified in the full model, 2 distinct dissociations are also identified: The significant path between personal experiences and trust that only emerges in the US model and the significant (and negative) path between familiarity and information corroboration that only emerges in the UK model, such that UK citizens are less likely to corroborate information if their primary source is familiar.

**Table 5:** The regression weights and critical ratio values for the main effects of the hypothesized model for UK participants.

Parameter	Unstandardized path coefficients	Critical ratio	*P* value
Credibility and impartiality → trust	0.912	10.982	<.001
Familiarity → trust	0.034	1.135	.26
PEX^a^ → trust	−0.031	−0.985	.33
Information corroboration → trust	0.029	1.141	.25
Credibility and impartiality → information corroboration	0.740	7.094	<.001
Familiarity → information corroboration	–0.139	–2.586	.01
PEX information corroboration	0.065	1.337	.18
Trust → coping	2.213	10.716	<.001
Trust → intention to act	0.782	9.929	<.001
Coping → intention to act	0.019	1.259	.21
Information corroboration → intention to act	0.058	1.656	.02

^a^PEX: personal experiences.

## Discussion

### Principal Findings

In terms of identifying the key predictors of trust and intention to act on health information, we found that trust significantly influenced self-reported intention to act on advice. Of the trust predictors, only credibility and impartiality was found to have a significant, direct relationship with trust. The effects of other variables (familiarity, personal experiences, and privacy) may be indirect and mediated through the other trust variables. For the role of personal experiences, it was found to have a significant direct effect on information corroboration, which in turn significantly predicted trust. Trust in turn was found to significantly relate to coping perceptions and intention to act on the advice. These results lead us to make the following observations.

The first point to note is that trust judgments significantly influence self-reported intention to act upon the health advice given online and furthermore, that these trust judgments reflect the extent to which people feel that the information sources are (1) credible, that is, contain good quality, relevant information, (2) well designed and presented, and (3) impartial, that is, contain information offered in the health consumer’s best interest. These results resonate with recent findings in the existing literature (see Sbaffi and Rowley [40] for a systematic review). For example, trust is known to predict intention to act upon health advice (eg, [21,41]). The relevance, quality, usefulness, and accuracy of information are known determinants that the information content is trustworthy [42]. The presentation, ease of use, and clarity of information are linked to perceptions of *professionalism* that, again, underpin judgments of trust [4,43,44] and, finally, the beliefs about objectivity and impartiality of the source also ensure trust [21].

**Figure 1:**
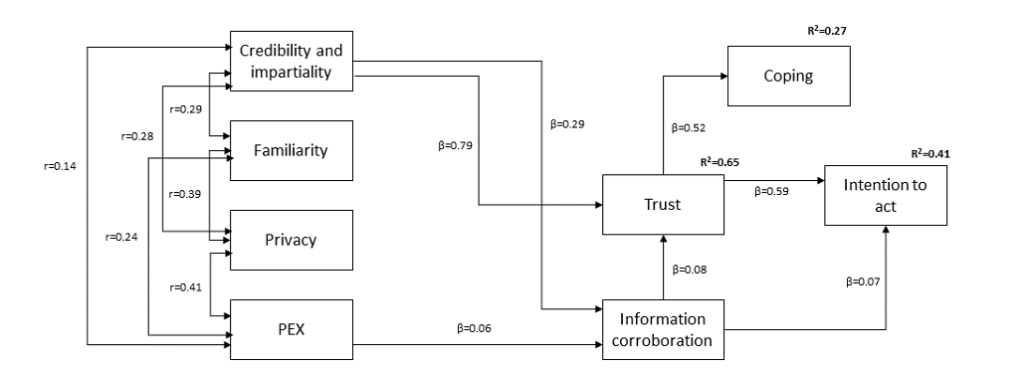
The trust model with significant standardized path coefficients. PEX: personal experiences.

Looking in more detail at the model presented in Figure 1, we can see interesting similarities and differences between the current model and the model developed 10 years ago [21]. Specifically, Harris et al [21] showed that 2 website factors (information quality and impartiality) directly influenced trust. In the model we present here, these same 2 website factors, now combined into 1 construct named *credibility and impartiality* are the strongest predictors of trust. Harris et al [21] also showed that trust and its relationship to intention to act were moderated by 2 cognitive processes—involving threat appraisal and information corroboration. They also, along with personal experiences factor, significantly affect the processes of information corroboration, which in turn affect both trust and intention to act upon the health advice given, with a final *coping* factor also moderating the relationship between trust and intention to act. In short, credibility of information and impartiality occupy pivotal roles in our decision to trust the information we view online, as it did in the earlier Harris et al model [21], something entirely consistent with the ways in which patients come to develop trust relationships with their physician where there is a strong belief that doctors act in the patients’ best interest (eg, [45]). Credibility and impartiality are key to trust in eHealth in 2019 as they were 10 years ago.

Online health information is also important in helping people to cope with health issues. When people trust sites that provide positive information about controlling symptoms or disease, it appears to help boost their overall sense of coping and efficacy. Although the model developed by Harris et al [21] did not find a significant relationship between trust and coping, we found that trust could account for 27% of the variance in coping. Our model confirms that placing trust in health websites is important in helping users to cope with their health issues, and this is in line with previous research indicating that seeking health information is in itself an important coping mechanism in enhancing adjustment to illness and in the promotion of health-related activities [46,47]. These findings could reflect a general improvement in the ways in which *trusted* sites offer information and advice and is possibly related to the rise of health websites offering patient experiences. We know from these data that patient experiences can influence trust (see below), and from the published literature, we know that personal experiences can also help people to feel supported in their health issues [18], but the ways that such experiences might directly affect coping would require further investigation.

The role of personal experiences in relation to trust is an interesting finding, and our contribution here is novel. As we noted earlier, one of the biggest changes to the internet is the sharing of patient stories and experiences. We note in our model that personal experiences can influence trust but only indirectly through first, influencing those judgments of credibility and impartiality that are so important in predicting trust and second, influencing the ways that people choose to corroborate the information they view online. This finding resonates with the idea that although personal experiences are often liked, they are not necessarily trusted automatically [23]. The literature concerning trust in ecommerce and, in particular, social commerce provides a useful reference point for considering the relationship between trust in personal experiences and trust in the health website overall [48,49]. The trustworthiness of other customers on a website can be transferred to the community and thus help build stronger confidence or trust in the website as a whole [50]. Similarly, on social media sites, high levels of trust in other site members lead to higher levels of trust in and use of the site as a whole [51].

The corroboration point is interesting as in the combined UK and US data we found that low information credibility and impartiality, as well as the presence of personal experiences, led to higher levels of corroboration but that the need for corroboration, sometimes referred to as *triangulation*, differed between the US and UK samples. Specifically, in the United Kingdom, if the primary source of information was familiar, then patients expressed less need to corroborate that information. This could well be a function of the dominance of the National Health Service as a single trusted health care provider in the United Kingdom (indeed, most UK respondents cited the National Health Service website as their source of health information), as opposed to the more complex marriage of public and private insurance–based systems operating in the United States, where WebMD was the most popular online choice. It may also be possible that the difference could lie in the extent to which advertising was present in the most popular websites, but our single item on advertising was insufficient to provide good data here. These results do resonate with the data provided by Schneider et al [38] who compared eHealth search patterns in a private (United States) and public (United Kingdom) health care market and concluded that the US system incentivizes personal search into eHealth and that free access to health care professionals in the United Kingdom (including telephone support) reduces the incentive to search widely for health information online.

The health corroboration process relies upon people being able to make an appropriate distinction between the more or less reliable sources of information they find online, and of course people may differ in their ability to make this distinction and to retain it when trying to recall information at a later date. The extent to which individuals engage in information corroboration is likely to reflect eHealth literacy and suggest that we may need to think more carefully about how to support different individuals when making trust judgments about online health information [14,52]. This may be particularly important when personal experiences are present as we know that personal experiences can help trigger a homophily “patients like me” response that may mean individuals are yet more vulnerable to targeted messages [13].

Health privacy was introduced as a factor in this study. It did not impact directly on trust in eHealth information, but the effect of privacy may be indirect and mediated through other trust variables. The data for this study were collected before the introduction of new privacy and data protection legislation that regulates the storage of personal data. The General Data Protection Regulation in Europe, which came into force in May 2018, is designed to harmonize data protection law across Europe and to bring the law up to date with technological advancements, specifically the increasing use of digital data. It would be interesting to see how a more transparent and direct message about data processing may impact on people’s perceptions of data privacy with regard to health websites going forward, and in the wake of increasing public concerns about the privacy of their health data, it is interesting to note new models that speculate on the role of health privacy in eHealth [53].

### Limitations and Future Work

Here we focus on a sample of the US and UK population, which limits how representative the findings are to other countries and cultures. Nevertheless, the model demonstrates the impact of trust in eHealth on health decision making for 2 different westernized countries (with different national health practices) and where use of the internet and technology is widespread. However, further work is required to explore country and demographic differences such as the growing role of information credibility skills in navigating online information [54]. Second, future work would benefit from assessing the impact of advertising using a more comprehensive range of items.

We speculated that corroboration across online sources may be linked to advertising, but the single *advertising* item within the general trust questionnaire did not load onto any of the factors in the model. It may still be worth exploring this relationship in future work as it may point to a changing and increasingly complex situation concerning the form and presence of advertising on health websites. Advertising comes in many forms, from banners to embedded endorsements. Pharmaceutical sites offer a holistic form of advertising, although some sites may choose to advertise through the use of crafted personal testimonials. The single item assessing advertising may be too blunt an instrument to detect attitudes toward these different commercial approaches and limits what we can deduce about the effect of advertising on trust in eHealth. The work is underway to assess people’s understanding of *advertising* more broadly in this context, especially given the blending of information sources in an online health care context [55].

### Conclusions

In conclusion, despite the large increase in new providers, new formats, and platforms, impartiality continues to remain a key predictor of trust in health websites as well as the extent to which users consider information sources to be credible. The presence of personal experiences information can have a positive influence on trust provided that users corroborate the information through additional sources.

## Abbreviations

AGFI: adjusted goodness-of-fit
CFI: comparative fit index
eHealth: electronic health
GFI: goodness-of-fit index
PCA: principal component analysis
RMSEA: root mean square error of approximation
SEM: structural equation modeling
